# Exogenous Application of Proteoglycan to the Cell Surface Microenvironment Facilitates to Chondrogenic Differentiation and Maintenance

**DOI:** 10.3390/ijms21207744

**Published:** 2020-10-19

**Authors:** Teruaki Masutani, Shuhei Yamada, Akira Hara, Tatsuji Takahashi, Paul G Green, Masayuki Niwa

**Affiliations:** 1 Medical Education Development Center, Gifu University School of Medicine, 1-1 Yanagido, Gifu City 501-1194, Japan; masutani-teruaki@ichimaru.co.jp; 2 Research & Development Dept., Ichimaru Pharcos Co., Ltd., 318-1 Asagi, Motosu City 501-0475, Japan; takahashi-tatsuji@ichimaru.co.jp; 3 Department of Pathobiochemistry, Faculty of Pharmacy, Meijo University, 150 Yagotoyama, Tempaku-ku, Nagoya 468-8503, Japan; shuheiy@meijo-u.ac.jp; 4 Department of Tumor Pathology, Gifu University Graduate School of Medicine, Gifu City 501-1194, Japan; ahara@gifu-u.ac.jp; 5 Department of Oral and Maxillofacial Surgery, UCSF, San Francisco, CA 94143, USA; Paul.Green@ucsf.edu

**Keywords:** osteoarthritis, cartilage, proteoglycan, aggrecan, extracellular matrix, pericellular matrix

## Abstract

Osteoarthritis (OA), a disease that greatly impacts quality of life, has increasing worldwide prevalence as the population ages. However, its pathogenic mechanisms have not been fully elucidated and current therapeutic treatment strategies are inadequate. In recent years, abnormal endochondral ossification in articular cartilage has received attention as a pathophysiological mechanism in OA. Cartilage is composed of abundant extracellular matrix components, which are involved in tissue maintenance and regeneration, but how these factors affect endochondral ossification is not clear. Here, we show that the application of aggrecan-type proteoglycan from salmon nasal cartilage (sPG) exhibited marked proliferative capacity through receptor tyrosine kinases in chondroprogenitor cells, and also exhibited differentiation and three-dimensional structure formation via phosphorylation of Insulin-like Growth Factor-1 Receptor and Growth Differentiation Factor 5 expression. Furthermore, sPG inhibited calcification via expression of Runx2 and Col10 (factors related to induction of calcification), while increasing Mgp, a mineralization inhibitory factor. As a result of analyzing the localization of sPG applied to the cells, it was localized on the surface of the cell membrane. In this study, we found that sPG, as a biomaterial, could regulate cell proliferation, differentiation and calcification inhibition by acting on the cell surface microenvironment. Therefore, sPG may be the foundation for a novel therapeutic approach for cartilage maintenance and for improved symptoms in OA.

## 1. Introduction

Life expectancy is increasing worldwide, and since osteoarthritis (OA) risk increases with age, there is a rapidly growing number of individuals with OA. Approximately 9.6% of men and 18% of women over the age of 60 have OA [1], with ~80% of individuals with OA reporting that this disease limits their movement and/or ability to exercise, and ~25% of those with OA have significantly impaired quality of life [1]. OA is a chronic disease in which joint deformity occurs due to excessive mechanical stress and age-related joint changes [2,3,4], but detailed molecular pathophysiological mechanisms that lead to the development of OA have not been elucidated, hindering development of effective treatments.

Cartilage tissue is composed of abundant extracellular matrix (ECM) and chondrocytes. In particular, articular cartilage ECM contributes to viscoelasticity that can withstand high cyclic loads [5]. It has been reported that an increasing Osteoarthritis Research Society International score has reduced viscoelastic properties to withstand compressive loads [6]. Chondrocytes, derived from mesenchymal chondroprogenitor cells, differentiate by expressing factors such as SRY-box transcription factor 9 (SOX9) and bone morphogenetic protein (BMP) through cell condensation by insulin-like growth factor 1 (IGF-1) [7,8,9], and once differentiated, chondrocytes produce type II collagen (COL2) and aggrecan, the major cartilage-specific proteoglycan [10,11]. Recently, it has been suggested that a decrease in expression level of the growth differentiation factor 5 (GDF5/BMP14) is closely related to the onset of OA [12,13]. GDF5 has also been investigated as an important regulator of chondrogenic differentiation as the member of transforming growth factor beta (TGF-β) superfamily [14,15]. Chondrocytes express runt related transcription factor 2 (Runx2), an essential regulator for chondrocyte maturation, which subsequently produces type X collagen (COL10) to be its target factor and matures into hypertrophic chondrocytes [16,17,18]. This process of terminal differentiation through matrix calcification is called endochondral ossification [16]. During the development of OA, abnormal hypertrophy of cartilage and mineralization of abnormal substrate due to endochondral ossification occur [19,20], and this stage is characterized by the formation of osteophytes [21]. This enhanced endochondral ossification is closely correlated with the progression of OA [16]. Parathyroid hormone-related protein (PTHrP), and its cognate receptor (PTHR), are involved in suppression of chondrocyte hypertrophy and in the maintenance of cartilage [22,23,24], and Matrix Gla Protein (MGP) contributes to suppression of mineralization [25,26].

In articular cartilage, activation of epidermal growth factor receptor (EGFR) in the uppermost superficial zone where chondroprogenitor cells are present, is important for tissue homeostasis [27]. At early stages of OA, tissue degeneration and destruction occur from the uppermost superficial zone of the cartilage, and ECM components, such as proteoglycan, are markedly decreased [19,28]. Aggrecan, which is constituted with chondroitin sulfate (CS) as the main glycosaminoglycan (GAG), contributes to joint viscoelasticity [28,29]. However, apart from its viscoelastic properties, the functional roles of aggrecan are still unclear. Recently, novel functions of ECM have been proposed, for example syndecan, a heparan sulfate proteoglycan in the central nervous system, not only provides physical support, but is also involved in induction of cell proliferation and differentiation, through the receptor tyrosine kinases [30,31,32]. In addition, the pericellular matrix (PCM) has a regulatory role in the cell surface microenvironment and cell signal regulation [33,34]. We hypothesize that enhanced matrix-degradation on the cell surface in OA is associated with deterioration of the cartilage environment, leading to abnormalities in cartilage metabolism. Even if the enhanced matrix degradation could be inhibited, OA may still progress unless the already disturbed cell surface microenvironment is improved.

The mechanisms by which ECM constituent components affect the process of endochondral ossification are not well known. Hyaluronan (HA) has been believed to exert is therapeutic effect due to its viscoelastic properties [35,36], while glucosamine and CS, constituent factors of GAG, have protective roles in cartilage due to their anti-inflammatory actions [37]. But, how aggrecan affects endochondral ossification is unknown. Since it has become possible to purify aggrecan-type proteoglycan from salmon nasal cartilage (sPG) with high precision, a clearer understanding of the effects of aggrecan′s functions have been elucidated [38,39]. In this study, we investigated the functional effect of sPG on cartilage by using chondroprogenitor cells ATDC5, a well-established in vitro model of endochondral ossification [40,41]. We also evaluated HA, collagen, CS and glucosamine hydrochloride (GlcN) in this in vitro model. In addition, we investigated whether sPG could be an effective therapeutic compound to treat OA by evaluating the mechanism of effectiveness of proteoglycan for each process of endochondral ossification.

## 2. Results

### 2.1. Effect on the Proliferation of ATDC5 Cells by Addition of ECM Constituting Factor

**Figure 1:**
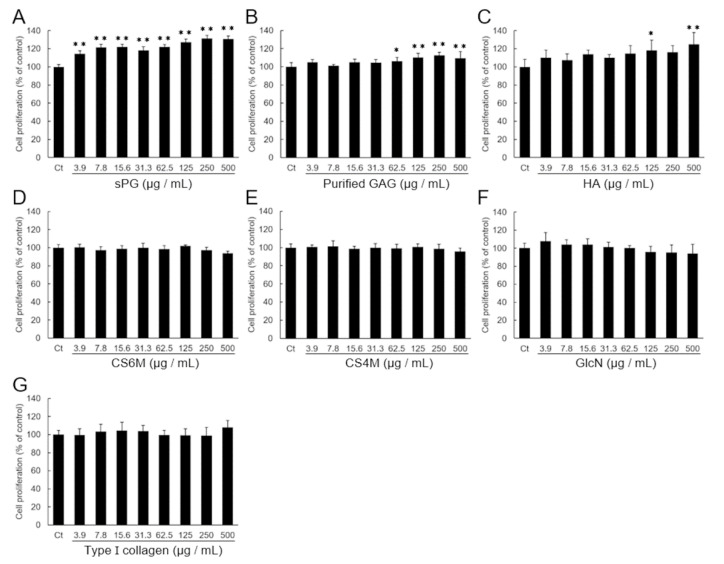
Comparison of cell proliferative effect in extracellular matrix (ECM) components on chondroprogenitor cells ATDC5. Cell growth analysis on the ECM components: aggrecan-type proteoglycan from salmon nasal cartilage (sPG) (**A**), purified GAG (**B**), Glycosaminoglycan from sPG, hyaluronan (HA) (**C**), CS6M (**D**), where 6 sulfate of chondroitin is the major, CS4M (**E**), where 4 sulfate of chondroitin is the major, glucosamine hydrochloride (GlcN) (**F**) and type I collagen (**G**). Proliferative ability of ATDC5 cells was assessed by the WST-8 assay after culturing for 96 h adding to each ECM component (3.9–500 μg/mL) under DME/F12 medium containing 0.5% FBS without insulin at 37 °C in a humidified atmosphere of 5% CO_2_ in air. Control (Ct) is without ECM component. These data are presented as the mean ± S.D. of six replicates. Significant differences show * *p* < 0.05; ** *p* < 0.01 vs. 0 μg/mL as control, Dunnett′s test.

ATDC5 cells were used to evaluate cell proliferation on undifferentiated chondroprogenitor cells. GAG preparation was separated and purified from the same lot number of sPG. The disaccharide composition of commercial CS and purified GAG preparations was confirmed by High Performance liquid chromatography (HPLC) (Supplementary Table S1). One of the two commercial CS preparations used in this study was mainly 6-sulfate (CS6M), and the other was 4-sulfate (CS4M). In the proliferation test, sPG, HA, CS6M, CS4M, GlcN or type I collagen was added after lowering the serum concentration to 0.5%, cultured for 96 h, and then evaluated by the WST-8 cell proliferation assay method. As shown in Figure 1A, a significant cell proliferation effect was observed after addition of sPG, in a dose-dependent manner. In addition, a significant proliferative effect was also observed in purified GAG, but its effect was low compared to the effect of sPG (Figure 1B). HA produced a small proliferative effect (Figure 1C). CS6M, CS4M, GlcN and type I collagen had no effect on cell proliferative activity (Figure 1D–G), and no cytotoxicity was observed with any of the ECM factors.

### 2.2. Effect of sPG on Cellular Proliferation-Related Receptor Activation

**Figure 2:**
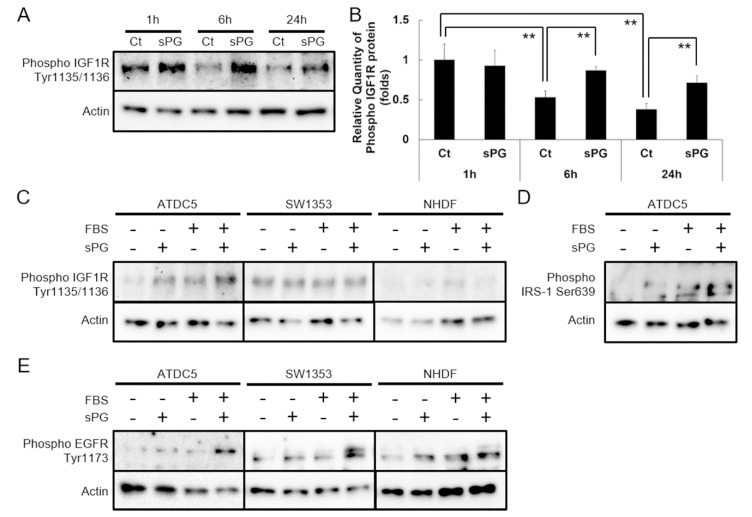
Effects of sPG on phosphorylation of Insulin-like Growth Factor-1 Receptor (IGF-1R) and Epidermal Growth Factor Receptor (EGFR) in cell proliferation. (**A**) Immunoblotting analysis on phosphorylation of IGF-1R in cell proliferation. ATDC5 cells were treated without (Ct) or with sPG (100 μg/mL) under DME/F12 medium containing 0.5% FBS without insulin for 1, 6, 24 h at 37 °C in a humidified atmosphere of 5% CO_2_ in air and harvested. Phosphorylation of IGF-1R were analyzed by immunoblotting with phospho-IGF-1R antibody. (**B**) Densitometric analysis of phospho-IGF-1R bands. The obtained bands were densitometrically analyzed by Image J and normalized relative to β-actin, as shown in the graph. Effects of sPG on phosphorylation of IGF-1R (**C**) and EGFR (**E**) in different cell types. ATDC5, SW1353 and NHDF cells were treated without or with sPG (100 μg/mL) under each medium containing FBS-free or 0.5% FBS for 24 h at 37 °C in a humidified atmosphere of 5% CO_2_ in air and harvested. Phosphorylation of IGF-1R or EGFR was analyzed by immunoblotting with phospho-IGF-1R antibody or with phospho-EGFR antibody. Phosphorylation of insulin receptor substrate-1 (IRS-1) (**D**) with ATDC5 was analyzed by immunoblotting with phospho-IRS-1 antibody. These data are presented as the mean ± S.D. of three independent experiments. Significant differences show ** *p* < 0.01; Tukey′s test.

In order to investigate the mechanism of cell proliferation shown in Figure 1, we examined the role of IGF-I signaling, which is known to be involved in the proliferation of mesenchymal cells, including chondroprogenitor cells. We observed changes in tyrosine phosphorylation of IGF-I receptor (Figure 2A). Phosphorylation was markedly reduced at 6 and 24 h by low serum without sPG, but phosphorylation was not reduced by addition of sPG at either 6 or 24 h (Figure 2B). We evaluated whether IGF-1R phosphorylation was increased by the addition of sPG in ATDC5 cells of the serum-free state, and whether sPG further increases phosphorylation of IGF-1R slightly induced by low serum. In serum-free medium, there was minimal phosphorylation of IGF-1R, but following addition of sPG, IGF-1R phosphorylation was significantly increased (Figure 2C). Modest IGF-1R phosphorylation was observed with low serum, and phosphorylation was further increased by addition of sPG (Figure 2C). Therefore, we evaluated SW1353, another chondrogenic cell line, and found that IGF-1R was constitutively phosphorylated in SW1353 cells in the serum-free state, and little phosphorylation was observed following addition of sPG (Figure 2C). For comparison, the same evaluation was performed in Normal Human Dermal Fibroblasts (NHDF), which exhibited no IGF-1R phosphorylation with either addition of low serum or with the addition of sPG (Figure 2C). Next, we assessed whether changes in IGF-1R phosphorylation by sPG in ATDC5 cells affected the phosphorylation of its downstream insulin receptor substrate-1 (IRS-1). In serum-free medium, there was minimal phosphorylation of IRS-1, but following addition of sPG, IRS-1 phosphorylation was slightly increased (Figure 2D). Modest IRS-1 phosphorylation was observed with low serum, and phosphorylation was further increased by addition of sPG (Figure 2D). We also evaluated another receptor tyrosine kinase, Epidermal Growth Factor Receptor (EGFR), and observed that EGFR phosphorylation, barely detectable in serum-free conditions, was slightly increased following addition of sPG, and in low serum conditions, addition of sPG significantly increased EGFR phosphorylation (Figure 2E). Interestingly, unlike IGF-1R, similar results were obtained in EGFR phosphorylation in ATDC5, SW1353 and NHDF cells (Figure 2C,E).

### 2.3. Effects of ECM Constituents on Chondrogenic Differentiation in ATDC5 Cells

**Figure 3:**
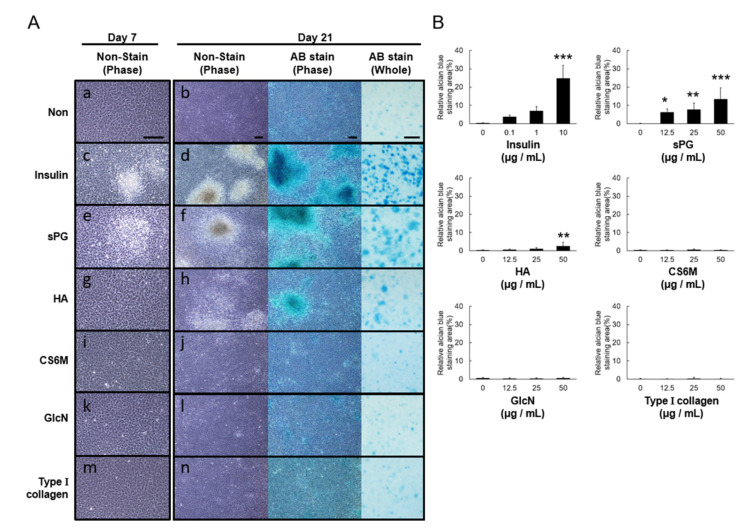
Comparison of chondrogenic differentiation effect in ECM components on ATDC5 cells. (**A**) Cell morphology of ATDC5 cells by phase-contrast micrographs. ATDC5 cells were then incubated in the absence (**a**) or the presence (50 μg/mL) of the matrix components sPG (**e**), HA (**g**), CS6M (**i**), GlcN (**k**) and type I collagen (m) under DME/F12 medium containing 5% FBS at 37 °C in a humidified atmosphere of 5% CO_2_ in air for another 7 days. Insulin (**c**) (10 μg/mL) was used as a positive control of chondrogenic differentiation induction. The cell morphology was observed with phase-contrast microscopy by non-stain Alcian blue (AB) staining of ATDC5 cells. ATDC5 cells were then incubated in the absence (**b**) or the presence (50 μg/mL) of the ECM components sPG (**f**), HA (**h**), CS6M (**j**), GlcN (**l**) and type I collagen (**n**) for 21 days from post-confluence. Cells were stained with 0.1% Alcian blue in 0.1 M HCl overnight. Cell morphology images before staining and Alcian blue-stained images on day 21 were observed with phase-contrast microscopy. The whole images were acquired with a digital camera. Insulin (**d**) (10 μg/mL) was used as a positive control. (**B**) Image quantitative evaluation of chondrogenic differentiation. The obtained whole images were quantified by WinROOF software. These data are presented as the mean ± S.D. of three independent experiments. Significant differences show * *p* < 0.05; ** *p* < 0.01; *** *p* < 0.001 vs. 0 μg/mL as control, Dunnett‘s test. Scale Bar = 200 μm (phase images), 3 mm (whole image).

We next evaluated the ability of ECM constituents to induce chondrogenic differentiation. After culture to confluence, changes in morphology were evaluated microscopically following addition of each ECM constitutive factor, in the absence of insulin. No change was observed in cells that did not have any additions to the culture medium (Figure 3A(a)), but a marked condensation of cells was observed 7 days after addition of sPG (Figure 3A(e)). No significant cell condensation occurred with any other ECM constitutive factor (Figure 3A(g, i, k, m)). Insulin, used as a positive control, produced marked cell condensation from day 5 after addition (Figure 3A(c)). Culturing continued until day 21, and chondrogenic differentiation potential was evaluated by formation of three-dimensional cartilage-like nodules and staining with Alcian blue. In cells that had no additions to the culture medium, there was no formation of three-dimensional cartilage-like nodules or Alcian blue staining (Figure 3A(b)). In contrast, treatment with insulin as a positive control (Figure 3A(d)) or sPG (Figure 3A(f)) produced prominent three-dimensional cartilage-like nodules and Alcian blue staining by phase and whole images. HA produced aggregation at day 21, but not three-dimensional cartilage-like nodule formation, and only weak Alcian blue staining (Figure 3A(h)). Neither CS6M, GlcN nor type I collagen produced cartilage-like nodule formation nor Alcian blue staining (Figure 3A(j,l,n)). Quantitative analysis of Alcian blue staining, performed using WinROOF Ver.6.3.1 software, showed that a sPG-induced increase in Alcian blue staining was concentration-dependent, and HA produced significantly increased staining at 50 μg/mL (Figure 3B). The other factors did not increase staining, while insulin produced a concentration-dependent increase (Figure 3B).

### 2.4. Evaluation of Three-Dimensional Structure in Cartilage-Like Nodules Induced by sPG in ATDC5 Cells

**Figure 4:**
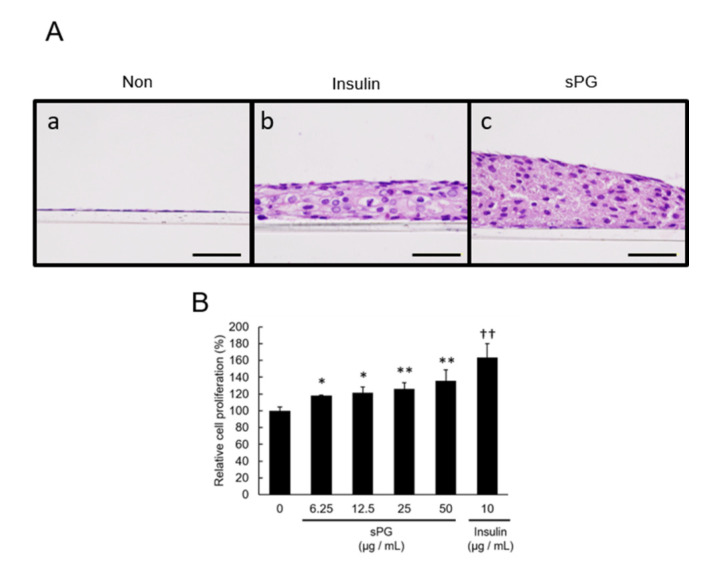
Formation evaluation on chondrogenic differentiated ATDC5 cells of sPG. (**A**) Histological observation of chondrogenic differentiated ATDC5 cells. Untreated cells (**a**) or cells treated with sPG (50 μg/mL) (**c**) or insulin (10 μg/mL) (**b**) were cultured on cell culture insert under DME/F12 medium containing 5% FBS at 37 °C in a humidified atmosphere of 5% CO_2_ in air for 21 days from post-confluence. Histological sections of cultured cells were stained with hematoxylin and eosin observed using phase-contrast microscopy. (**B**) Cell proliferative effect of sPG in chondrogenic differentiated ATDC5 cells. Proliferative ability of untreated cells or cells treated with sPG 6.25–50 μg/mL or insulin 10 μg/mL was assessed by the WST-8 assay. These data are presented as the mean ± S.D. of three independent experiments. Significant differences of sPG show * *p* < 0.05; ** *p* < 0.01 vs. 0 μg/mL as control, Dunnett′s test. Significant differences of insulin show †† *p* < 0.01 vs. 0 μg/mL as control, Student′s t-test. Scale Bar = 100 μm.

Regarding the cartilage-like nodule induced by the addition of sPG seen in Figure 3, further three-dimensional tissue sections were prepared and evaluated by hematoxylin and eosin (HE) staining. ATDC5 cells were firstly cultured to confluence on cell culture insert, then incubated for 21 days with or without sPG, or insulin as a positive control. Thereafter, paraffin sections of cells were prepared and HE-stained, and images were acquired under microscopy and analyzed. For cells with no additions to the medium, only monolayer chondroprogenitor cells were observed (Figure 4A(a)). Addition of insulin, however, induced differentiated multilayered structure, as well as cells with enlarged nuclei (Figure 4A(b)). Addition of sPG also induced a three-dimensional multilayered structure, but enlargement of the nuclei was less than that seen in insulin-treated cells (Figure 4A(c)). Of note, sPG induced a structural form that was multilayered, with round-shaped nuclei (Figure 4A(c)). In addition to induction of prominent cartilage-like nodule formation and three-dimensional differentiation, we also evaluated if sPG also concomitantly increased in the number of cells. Using the WST-8 assay, we observed that both sPG and insulin induced significant cell proliferation compared to cells without addition, suggesting that cell proliferation occurs in three dimensions (Figure 4B).

### 2.5. Effect of sPG on Chondrogenic Differentiation-Related Factor in ATDC5 Cells

**Figure 5:**
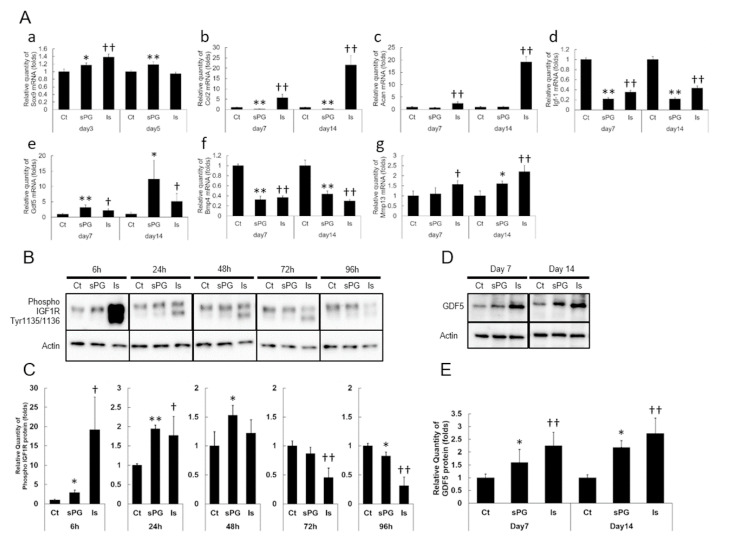
Effects of sPG on chondrogenic differentiation factors in ATDC5 cells. (**A**) Real-time quantitative polymerase chain reaction (PCR) analysis of mRNA expression on chondrogenic differentiation factors *Sox9*, *Col2a1*, *Acan*, *Igf-1*, *Gdf5*, *Bmp4* and *Mmp13*. Untreated cells (Ct) or cells treated with sPG (50 μg/mL) or insulin (Is) (10 μg/mL) were cultured under DME/F12 medium containing 5% FBS at 37 °C in a humidified atmosphere of 5% CO_2_ in air for 3–14 days from post-confluence. Relative expression levels of *Sox9* (**a**), *Col2a1* (**b**), *Acan* (**c**), *Igf-1* (**d**), *Gdf5* (**e**), *Bmp4* (**f**) and *Mmp13* (**g**) in ATDC5 treated without or with sPG or insulin was normalized to an endogenous control, *RPS18*. Significant difference of sPG on day 3 or 7 and day 5 or 14 show *p < 0.05; **p < 0.01 compared to 0 μg/mL as control, Student′s t-test. Significant differences of Insulin on day 3 or 7 and day 5 or 14 show † *p* < 0.05; †† *p* < 0.01 compared to 0 μg/mL as control, Student′s t-test. (**B**) Immunoblotting analysis on phosphorylation of IGF-1R in chondrogenic differentiation. ATDC5 cells were treated without (Ct) or with sPG (50 μg/mL) or insulin (Is) (10 μg/mL) under DME/F12 medium containing 5% FBS for 6, 24, 48, 72 and 96 h and harvested. Phosphorylation of IGF-1R was analyzed by immunoblotting with phospho-IGF-1R antibody. (**C**) Densitometric analysis of phosph-IGF-1R bands. The obtained bands were densitometrically analyzed by Image J and normalized relative to β-actin, as shown in the graph. Significant differences of sPG show * *p* < 0.05; ** *p* < 0.01 compared to 0 μg/mL as control and insulin show † *p* < 0.05; †† *p* < 0.01, Student′s t-test. (**D**) Immunoblotting analysis on GDF5 protein in chondrogenic differentiation. ATDC5 cells were treated without (Ct) or with sPG (50 μg/mL) or insulin (Is) (10 μg/mL) under DME/F12 medium containing 5% FBS for 7 and 14 days and harvested. GDF5 protein levels were analyzed by immunoblotting with GDF5 antibody. (**E**) Densitometric analysis of GDF5 bands. The obtained bands were densitometrically analyzed by Image J and normalized relative to β-actin, as shown in the graph. Significant differences of sPG on day 7 and day 14 show * *p* < 0.05; ** *p* < 0.01 compared to 0 μg/mL as control, Student′s t-test. Significant differences of insulin on day 7 and day 14 show † *p* < 0.05; †† *p* < 0.01 compared to 0 μg/mL as control, Student′s t-test.

Gene expression analysis was carried out by using the real-time quantitative polymerase chain reaction (PCR) method to determine what gene expression changes occur during chondrogenic differentiation in ATDC5 cells produced by sPG and insulin (as a positive control), compared to cells with no addition of sPG or insulin. *Sox9* mRNA expression was slightly increased after 3 days with insulin and after 5 days with sPG (Figure 5A(a)). Insulin also increased *Col2a1* and *Acan* mRNA expression, but in contrast, sPG suppressed expression of *Col2a1*, and had no effect on *Acan* (Figure 5A(b,c)). Both insulin and sPG markedly inhibited the mRNA expression level of *Igf-1* (Figure 5A(d)). The mRNA expression of *Gdf5* was significantly increased by the addition of insulin or sPG (Figure 5A(e)). Insulin and sPG significantly inhibited the mRNA expression of *Bmp4* (Figure 5A(f)). Insulin showed markedly increased mRNA expression of *Mmp13*, but the effect of sPG was lower than that of insulin (Figure 5A(g)). Since sPG formed Alcian blue-positive cartilage-like nodules through cell condensation, phosphorylation of IGF-1R was evaluated to investigate IGF-1 activation. sPG produced a marked increase in phosphorylation at Tyr1135/1136 of the IGF-1R compared to the non-addition condition (Figure 5B,C). Of note, sPG-induced increase in phosphorylation occurred between 6 and 48 h, but at 72 and 96 h, phosphorylation decreased to control levels (Figure 5B,C). By comparison, insulin produced marked phosphorylation at 6 h, which decreased over time, so that at 72 and 96 h, phosphorylation level was significantly lower than control (Figure 5B,C). Since we observed that sPG induced a marked increase in *Gdf5* mRNA expression, we next evaluated GDF5 protein expression, using a specific antibody, and observed that sPG increased GDF5 protein expression from day 7, with a significant increase in expression observed on day 14 (Figure 5D,E). In addition, insulin also produced a significant increase in GDF5 protein expression (Figure 5D,E).

### 2.6. Influence of ECM Constituting Factors on Cartilage Calcification in ATDC5 Cells

**Figure 6:**
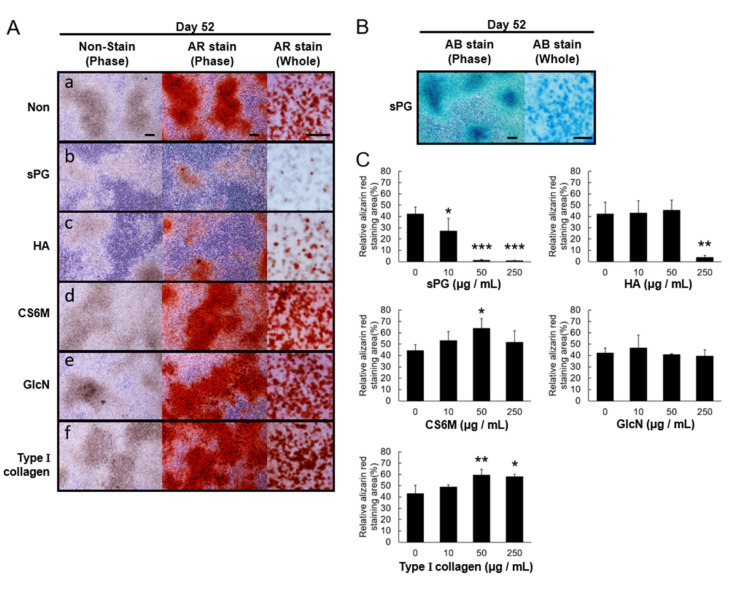
Comparison of inhibiting effects on cartilage calcification in ECM components on ATDC5 cells. (**A**) Alizarin Red (AR) staining of ATDC5 cells. ATDC5 cells were then incubated in the absence (**a**) or the presence (250 μg/mL) of the ECM components sPG (**b**), HA (**c**), CS6M (**d**), GlcN (**e**) and type I collagen (**f**) under αMEM medium containing 5% FBS and Insulin at 37 °C in a humidified atmosphere of 3% CO_2_ in air for 24 days from post-chondrogenic differentiation and stained with 1% Alizarin Red. Cell morphology images before staining and Alizarin Red-stained images on day 52 were observed with phase-contrast microscopy. The whole images were acquired with a digital camera. (**B**) Alcian Blue (AB) staining on day 52 of sPG (250 μg/mL) was confirmed. (**C**) Image quantitative evaluation on inhibition of cartilage calcification. The obtained whole images were quantified by WinROOF software. These data are presented as the mean ± S.D. of three independent experiments. Significant differences show * *p* < 0.05; ** *p* < 0.01; *** *p* < 0.001, Dunnett′s test. Scale Bar = 200 μm (phase images), 3 mm (whole images).

We assessed the ability of ECM constituting factors to inhibit cartilage calcification. ATDC5 cells were cultured to confluence, and then incubated for 21 days in the presence of insulin to induce cartilage-like nodules, and then incubated with ECM constituting factors under calcification-inducing conditions, until day 52, when cellular morphology and calcification were evaluated. In the control treatment group, cells incubated without additional factors, there was a decrease of light transmission of the cartilage-like nodule, presence of crystalline matter and a marked increase in staining by Alizarin red, a marker of calcification (Figure 6A(a)). In contrast, in cells incubated with sPG, there was no decrease in light transmission of cartilage-like nodules or crystalline matter formation, and the Alizarin red staining (quantitated using WinROOF Ver.6.3.1 software) was very weak (Figure 6A(b)). We also observed strong Alcian blue staining in sPG-treated cells on day 52 (Figure 6B). In addition, incubation with HA produced Alizarin red staining, but less than that in the control (no treatment) cells (Figure 6A(c)). CS6M, GlcN and type I collagen did not suppress the Alizarin red staining (Figure 6A(d–f)). Alizarin red-positive staining was suppressed by sPG from 10 μg/mL in a concentration-dependent manner, while HA produced significant suppression only at 250 μg/mL (Figure 6C). Neither GlcN, CS6M nor type I collagen suppressed Alizarin red staining, but the latter two induced calcification at 50 μg/mL, and at 50 and 250 μg/mL, respectively (Figure 6C).

### 2.7. Effect of sPG on Cartilage Calcification-Related Factor (mRNA)

**Figure 7:**
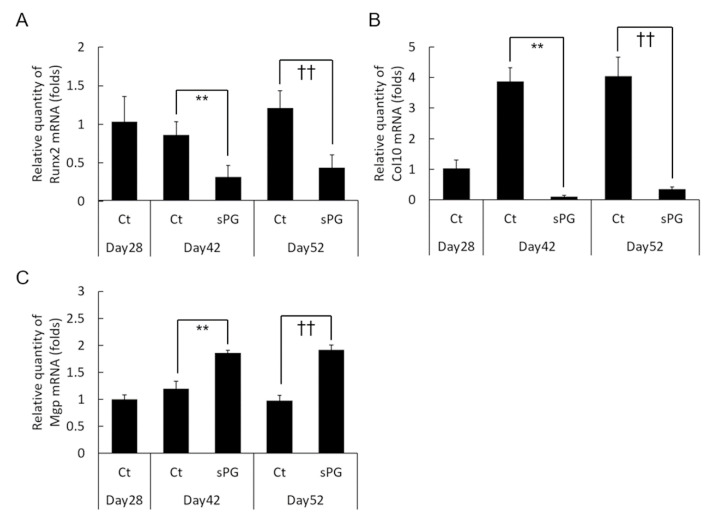
Effects of sPG on cartilage calcification factors in ATDC5 cells. Real-time quantitative PCR analysis of mRNA expression on calcification-related factors, *Runx2*, *Col10a1* and *Mgp*. Untreated cells or cells treated with sPG (50 μg/mL) were cultured under αMEM medium containing 5% FBS and insulin at 37 °C in a humidified atmosphere of 3% CO_2_ in air for 24 days from post-chondrogenic differentiation. Relative expression levels of *Runx2* (**A**), *Col10a1* (**B**) and *Mgp* (**C**) in ATDC5 treated without or with sPG were normalized to an endogenous control, RPS18. These data are presented as the mean ± S.D. of three independent experiments. Significant differences on day 42 show ** *p* < 0.01. Significant differences of day 52 show †† *p* < 0.01, Student′s t-test.

To investigate the mechanism of the potent inhibitory effect of sPG on calcification observed in Alizarin red experiments, we performed gene expression analysis in ATDC5 cells using real-time quantitative PCR. sPG significantly inhibited the expression of *Runx2*, a major cartilage calcification inducer (Figure 7A), and *Col10a1*, a Runx2 target and a cartilage calcification marker (Figure 7B). In contrast, sPG significantly increased the expression of *Mgp*, a suppressor of cartilage calcification (Figure 7C).

### 2.8. Influence of sPG on Continued Calcification Conditions

**Figure 8:**
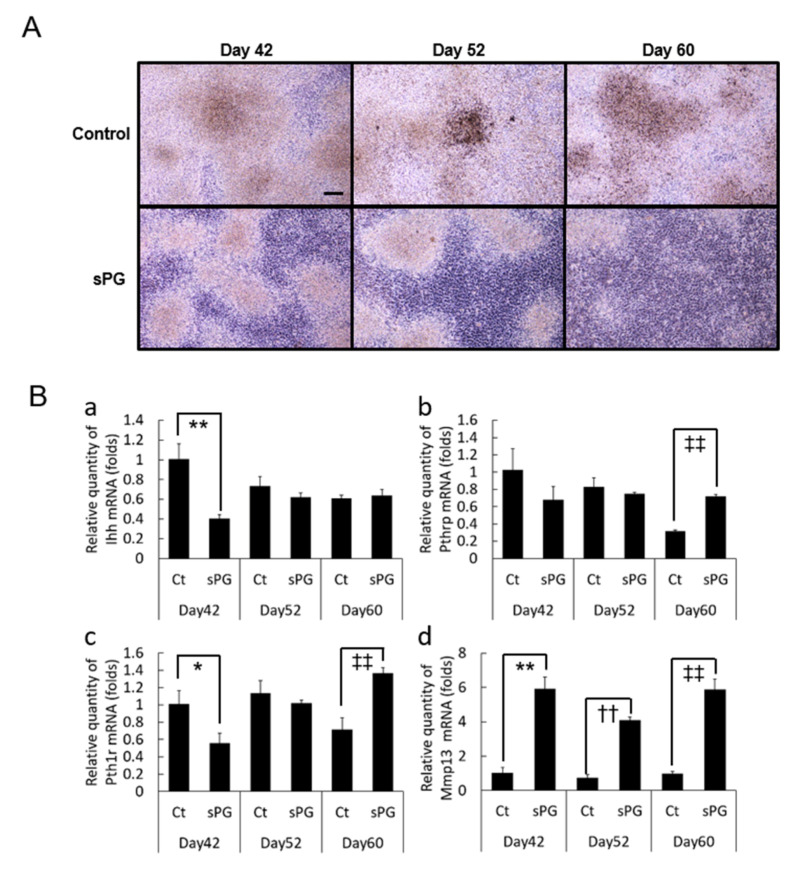
Effect of sPG on loss of calcified layer of ATDC5 cells. (**A**) Cell morphology of ATDC5 cells by phase-contrast micrographs. ATDC5 cells were then incubated in the absence (Ct) or the presence of sPG (50 μg/mL) under αMEM medium containing 5% FBS and insulin at 37 °C in a humidified atmosphere of 3% CO_2_ in air for 14, 24 and 32 days from post-chondrogenic differentiation (28 days). The cell morphology was observed with phase-contrast microscopy. (**B**) Real-time quantitative PCR analysis of mRNA expression on disappearance-related factors of calcified layer, *Ihh*, *Pthrp*, *Pth1r* and *Mmp13*. Untreated cells (Ct) or cells treated with sPG (50 μg/mL) were cultured under αMEM medium containing 5% FBS and insulin at 37 °C in a humidified atmosphere of 3% CO_2_ in on days 14, 24 and 32 from post-chondrogenic differentiation (28 days). Relative expression levels of *Ihh* (**a**), *Pthrp* (**b**), *Pth1r* (**c**) and *Mmp13* (**d**) in ATDC5 treated without or with sPG were normalized to an endogenous control, *RPS18*. These data are presented as the mean ± S.D. of three independent experiments. Significant differences on day 42 show * *p* < 0.05; ** *p* < 0.01, on day 52 show †† *p* < 0.01 and on day 60 show ‡‡ *p* < 0.01, Student′s t-test. Scale Bar = 200 μm.

Culturing was continued for 60 days under conditions of inducing cartilage calcification, to evaluate the effect of sPG on ATDC5 morphology. We observed that sPG decreased crystalline matter and cartilage calcification without changing the monolayer composed of undifferentiated chondroprogenitor cells (Figure 8A). Furthermore, the expression level of Indian hedgehog (*Ihh*, a bone morphogenetic factor) was significantly suppressed by sPG at day 42, but no inhibition was observed at day 60 (Figure 8B(b)). While sPG suppressed both *Pthrp* and *Pth1r* expression at day 42, it increased both at day 60 (Figure 8B(c)). However, unlike induction of chondrogenic differentiation, addition of sPG under calcification-inducing condition markedly increased expression of *Mmp13* (Figure 8B(d))

### 2.9. Localization Analysis of sPG Applied to Living Cells

**Figure 9:**
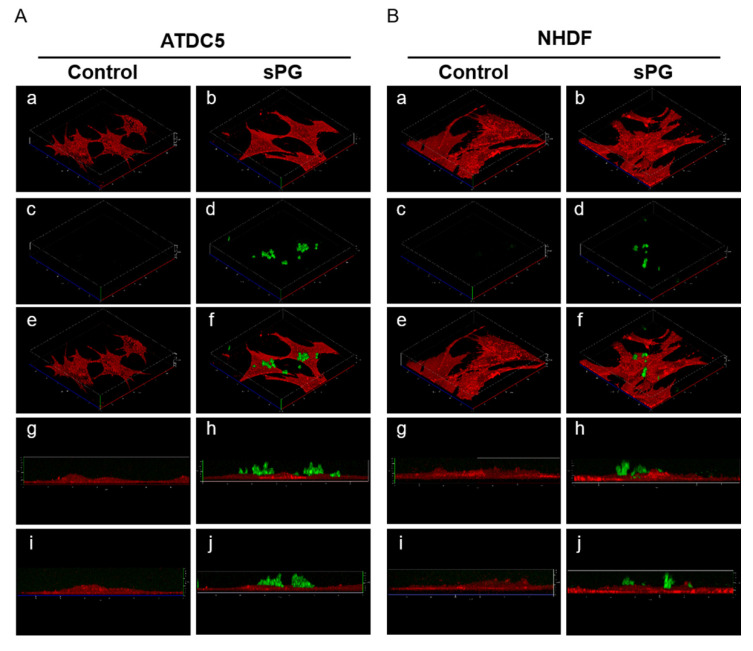
Three-dimensional live cell imaging of sPG localization using Confocal Laser Scanning Microscopy (CLSM). Three-dimensional localization analysis of sPG in ATDC5 cells (**A**) and NHDF cells (**B**). ATDC5 cells or NHDF cells were treated ATTO 488 alone (Control) or ATTO 488-labeled sPG for 30 min. After washing with PBS, each cell was treated with CellMask for 30 min. After each cell was also washed with PBS, optical sectioning of the live cells were acquired using CLSM at 24 h. In both A and B, (**a**–**f**) indicate the XYZ three-dimensional, (**g**,**h**) indicate the XZ-section, and (**i**,**j**) indicate the YZ-section. (**a**,**b**) indicate a cell membrane using CellMask, (**c**,**d**) indicate ATTO 488 alone or ATTO 488-labeled sPG, and (**e**–**j**) indicate each Merge.

The localization of the applied sPG was analyzed by live cell imaging. sPG were fluorescently labeled with ATTO 488, and cell membranes were labeled with CellMask. The fluorescently labeled sPG were applied to ATDC5 cells. Three-dimensional images were obtained using Confocal Laser Scanning Microscopy (CLSM) in live cells 1 and 24 h after the addition of sPG. As a result, it was confirmed that the sPG were adhered to the cell membrane surface (Figure 9A(a–j)). Furthermore, no change in localization was observed 1 and 24 h after sPG was added, and both showed localization on the membrane surface (Figure 9A(a–j)). Although sPG were localized on the cell membrane surface, no intracellular localization was observed. In addition, similarly to ATDC5, localization of sPG on the cell membrane surface was observed in NHDF, and no localization in cells was observed (Figure 9B(a–j)). As compared to the case where no sPG was added, no remarkable change was observed in the cell morphology, and no damage due to sPG adhesion was observed (Figure 9A,B).

## 3. Discussion

Cartilage is composed of abundant ECM, and proteoglycan, one of the constituent factors in ECM, contributes to cartilage viscoelasticity [5]. But, beyond proteoglycan′s viscoelasticity, little is known about its other functions. In OA, the ECM containing proteoglycan is decreased [42], and abnormalities in endochondral ossification, as well as reduction in viscoelasticity, have been reported [16]. Therefore, in this study, we applied sPG to cells at multiple stages of endochondral ossification to investigate possible novel cartilage regulatory functions for proteoglycan, which goes beyond its role in cartilage viscoelasticity.

Chondroprogenitor cells are present in the superficial zone of articular cartilage and are involved in the supply of chondrogenic differentiation. The superficial zone of cartilage is the first layer to be damaged in OA, and since chondroprogenitor cells possess only a weak capacity for repair [27,43], protecting or enhancing proliferation of chondroprogenitor cells in this layer is very important for ameliorating OA. In this study, sPG was found to have a significant cell proliferation effect, suggesting that it could be an effective candidate for maintaining chondroprogenitor cells. Purified GAG, glucosamine and CS, which are components of proteoglycan, had slight or no effect on cell growth, in contrast to sPG, which markedly affected cell growth, indicating that the whole proteoglycan structure containing core protein and GAG side chains is required. This effect of proteoglycan is believed to be mediated via activation of Extracellular Signal-regulated Kinase (ERK), but the relevant receptors have not been elucidated [39]. In this study, we observed that sPG acts via IGF-1R and EGFR to affect cell growth effect. This is consistent with the finding that activation of the EGFR signal in the outermost layer of cartilage is important for maintenance of cartilage and prevention of OA [27]. Therefore, induction of phosphorylation of EGFR by sPG may be an effective therapeutic approach in OA. Furthermore, proteoglycan acts via receptor tyrosine kinases, and syndecan, a heparan sulfate proteoglycan, regulates Fibroblast Growth Factor (FGF)-FGF Receptor activation in the central nervous system [31,44,45,46]. Decorin, a CS-type proteoglycan, regulates activation to EGF-EGFR and IGF-IGFR in the epithelial and muscular systems [47,48]. The present study provides new insights into the proliferative and differentiating mechanisms by proteoglycan on chondroprogenitor cells via the two types of receptor tyrosine kinase, IGF-1R and EGFR. 

The cartilage pericellular matrix (PCM) is not only a mechano-transducer, but it also transduces signals from growth factors and other regulatory factors via receptor tyrosine kinases [33]. OA progression is associated with changes in composition of PCM [34], for example, type I collagen in PCM is increased in OA [49]. We now show that application of type I collagen to cells also significantly increased calcification, suggesting that it promotes calcification via PCM. Recently, the addition of fluorescently labeled proteoglycan to cultured chondrocytes caused radial localization on the cell surface as a component of PCM and coated chondrocytes [50]. In this study, fluorescently labeled sPG showed adhesion to the cell membrane surface, suggesting that sPG regulates cell proliferation, differentiation and calcification inhibition through changes in the pericellular microenvironment. Furthermore, we hypothesize that the localization of sPG on the cell membrane surface contributes to the regulation of receptor tyrosine kinase activity. In OA, the suppression of degradation of cartilage constituent matrix by using anti-inflammatory and cytokine blocker was not sufficiently effective [51,52], since the cell surface microenvironment was not improved [33,34]. Therefore, in this study, the application of proteoglycan to cells may be a new approach to induce signals related to the induction and maintenance of chondrogenic differentiation by adhering to the cell membrane surface and changing the cell surface microenvironment. 

The balance between cartilage synthesis and degradation is disrupted in OA [53], suggesting that induction of chondrogenic differentiation is important in improving this balance. Addition of IGF-1 or BMP promotes chondrogenic differentiation [7,40,54], and we have shown that application of sPG, cell surface matrix, to cells stimulates IGF-1R and promotes the production of GDF5 (BMP14), thereby inducing chondrocyte differentiation. A decrease in expression level of GDF5 is associated with OA [12,13], and is attracting attention as an important factor in cartilage formation [14,55]. Mesenchymal condensation is a major feature in differentiation of chondroprogenitor cells into chondrocytes [56], and we observed that marked mesenchymal condensation was induced by sPG, suggesting that alteration of the pericellular microenvironment contributes to induction of the early differentiation. So far, IGF-1 has been implicated in early cell condensation [54], but sPG alone can enhance Tyr1135/1136 phosphorylation of IGF-1R, suggesting that the localization of sPGs on the cell membrane surface is related to cell condensation. In the present study, the significant decrease in Igf-1 mRNA expression levels upon addition of sPG or insulin may be due to feedback from IGF-1R phosphorylation. This suggests that the cell surface localization of sPG is partially involved in the functions of insulin and IGF-1. Furthermore, the significant reduction in Bmp4 expression by sPG or insulin may also be due to the feedback associated with increased protein expression of GDF5. However, hypertrophy of cells and nuclei in three-dimensional tissues was not observed in sPG-treated tissues.

While glucosamine and CS appear to have almost no effect in treating OA, HA does appear to have some therapeutic value [35,36]. Recently, HA has been reported to suppress the expression of Runx2 and have a CD44-mediated inhibitory effect on proinflammatory cytokine-induced ADAMTS4, thereby reducing cartilage calcification and potentially having a chondroprotective role [57,58]. In the present study, we observed inhibition of calcification by HA, as well as proliferation of chondroprogenitor cells and chondrocyte differentiation. However, none of these effects were observed for GlcN, CS or collagen. We showed here that sPG has superior effects on the proliferation of chondroprogenitor cells, induction of chondrocyte differentiation and inhibition of chondrocyte calcification. To our knowledge, these effects of proteoglycan have not previously been reported. In addition, sPG was significantly more effective than HA in the above-mentioned effects, suggesting that it could be a novel therapeutic for the treatment of OA. Regarding the inhibitory effect of sPG on calcification, it inhibited the expression of Runx2 and Col10, suggesting that the mechanism is similar to that of HA. The inhibitory effect of sPG on calcification via the induction of MGP has not been reported, and the results obtained here are novel findings. 

We also observed that addition of sPG and HA suppressed calcification, suggesting that reduction of proteoglycan and HA in OA leads to abnormally enhanced endochondral ossification and osteophyte formation. OA may be due to a number of pathophysiological processes, such as enhanced ECM degradation, abnormal cell signal transduction and abnormal endochondral ossification, to produce osteophytes [16]. Aggrecan is altered or reduced in OA [19,28], suggesting that aggrecan itself is closely related to abnormalities in cartilage metabolism as well as a decrease in viscoelasticity. In addition, in aggrecan-deficient mice, abnormalities in cartilage ECM as well as structural and metabolism occur [59,60]. Thus, it is thought that ECM degradation in OA not only decreases the cartilage viscoelasticity, but also worsens the cartilage metabolic environment, leading to complex symptoms of OA. In addition, even if ECM degradation is inhibited with anti-inflammatory drugs, the lost ECM is not restored, and deterioration of cartilage metabolic environment continues, allowing the progression of OA. Therefore, treatment with aggrecan-type proteoglycan is a novel therapeutic approach for ECM supplementation, which could also improve the metabolic environment of cartilage.

Addition of PTH reduces cartilage-like nodules and changes them into flat cells, and suppresses chondrocyte hypertrophy and calcification [40,41], which is similar to the changes observed with the application of sPG. In addition, the expression of Col10 was suppressed by the addition of PTH, being similar to the previous reports [61]. As described above, it has been suggested that PTH de-differentiates chondrocytes into an undifferentiated state. In this study, we hypothesize that Pthrp and Pth1r were induced by changes in the cell surface microenvironment by application of sPG. In mouse experiments, PTH contributes to cartilage maintenance, thereby suppressing cartilage degeneration [62], and a similar effect is expected for proteoglycan. Degradation of proteoglycan occurs during cartilage calcification [41], and future studies will reveal whether similar results occur when the degradation of proteoglycan is inhibited during induction of calcification. This suggests that endogenous proteoglycan in the cartilage may be involved in cartilage maintenance and regulation, thereby indicating that cartilage improvement may be achieved by changing the pericellular microenvironment with supplementation of proteoglycans.

## 4. Materials and Methods 

### 4.1. Materials

sPG is a product extracted with 4% acetic acid and subsequently purified, as we have previously reported [63]. D-(+)-glucosamine hydrochloride and Type I collagen were purchased from FUJIFILM Wako Pure Chemical Corporation, Osaka, Japan. HA was purchased from Shiseido Co., Ltd., Tokyo, Japan. Chondroitin sulfate sodium was purchased from Tokyo Chemical Industry Co., Ltd., Tokyo, Japan and Kishida Chemical Co., Ltd., Osaka, Japan. Glycosaminoglycan (GAG) was purified from sPG. The GAG and CS were digested with chondroitinase ABC. The HPLC analysis of the disaccharide composition was carried out under the following conditions: column, YMC-Pack PA-G (150 × 4.6 mm, YMC Co., Inc., Kyoto, Japan), flow rate, 0.5 mL/min, solvent, 1 M NaH_2_PO_4_ and 16 mM NaH_2_PO_4_, gradient, 16 mM to 1 M over 60 min, detection, UV absorption at 232 nm.

### 4.2. Cells and Cell Culture 

A chondroprogenitor cell line, ATDC5, was purchased from the RIKEN Cell Bank, Japan. ATDC5, as chondroprogenitor cells, were cultured in DMEM/Ham′s F12 containing 5% fetal bovine serum (FBS), penicillin/streptomycin, 10 µg/mL transferrin (Sigma-Aldrich, St. Louis, MO, USA) and 3 × 10^−8^ M sodium selenite (Sigma-Aldrich, St. Louis, MO, USA) at 37 °C in a humidified atmosphere of 5% CO_2_ in air. SW1353 cells, purchased from the Japanese Collection of Research Bioresources Cell Bank, Osaka, japan and American Type Culture Collection (ATCC), Manassas, VA, USA, were cultured in DMEM/Ham′s F12 containing 10% FBS and penicillin/streptomycin at 37 °C in a humidified atmosphere of 5% CO_2_ in air. Normal Human Dermal Fibroblasts (NHDF) cells were purchased from Kurabo industries Ltd., Osaka, Japan. NHDF cells were cultured in DMEM containing 10% FBS and penicillin/streptomycin at 37 °C in a humidified atmosphere of 5% CO_2_ in air. All of the above cells were medium exchanged once every 2 or 3 days.

### 4.3. Cell Proliferation Assay

Cell proliferation was assessed according to the manufacturer′s instruction for cell counting kit (CCK-8; Dojindo, Kumamoto, Japan), together with the cell viability assay, WST-8. Proliferation of chondroprogenitor cells was evaluated using the CCK-8 Kit after culturing for 96 h after adding each ECM component (3.9–500 μg/mL), under DME/F12 medium containing 0.5% FBS, penicillin/streptomycin, 10 µg/mL transferrin and 3 × 10^−8^ M sodium selenite at 37 °C in a humidified atmosphere of 5% CO_2_ in air without insulin on ATDC5 cells. Control cells were without addition of ECM components. 

Cell proliferation during chondrogenic differentiation was evaluated after culturing for 21 days after adding sPG (6.25–50 μg/mL) or insulin (10 μg/mL) under DME/F12 medium containing 5% FBS, penicillin/streptomycin, 10 µg/mL transferrin and 3 × 10^−8^ M sodium selenite at 37 °C in a humidified atmosphere of 5% CO_2_ in air on ATDC5 cells. Control was without sPG and insulin. The medium containing sPG or insulin was changed once every 2 or 3 days. The medium containing no sPG or insulin as control was also changed once every 2 or 3 days.

### 4.4. Immunoblotting

Cells were lysed with radioimmunoprecipitation assay (RIPA) buffer after washing with cold phosphate-buffered saline (PBS) and total protein was extracted. The cellular debris was removed from the extracted protein by centrifugation for 5 min after freezing and thawing. The BCA method (Thermo Fisher Scientific, Waltham, MA, USA) was used to test protein concentration. The obtained proteins were eluted by boiling in 2x sodium dodecyl sulfate (SDS) sample buffer, including 2-mercaptoethanol for 5 min. The samples were separated by SDS-polyacrylamide gel electrophoresis (PAGE), followed by protein blotting onto a polyvinylidene fluoride (PVDF) membrane using Trans-Blot Turbo Transfer System (Bio-Rad Laboratories, Hercules, CA, USA). The protein-blotted membranes were blocked with PVDF Blocking Reagent (Toyobo, Osaka, Japan) for 1 h at room temperature. They were then incubated with anti-phospho-IGF1R Tyr1135/1136 antibody (Cell Signaling Technology, Beverly, MA, USA) at 1:1000 dilution, anti-phospho-IRS-1 Ser639 antibody (Elabscience Biotechnology, Houston, TX, USA) at 1:1000 dilution, anti-phospho-EGFR Tyr1173 antibody (Sigma-Aldrich, USA) at 1:1000 dilution and anti-GDF 5 antibody (Abcam, Cambridge, UK) or anti-β-actin antibody (Sigma-Aldrich, USA) at 1:10,000 dilution in CanGet Signal Solution 1 (Toyobo, Osaka, Japan) overnight at 4 °C. After washing three times for 5 min with TBS-Tween 20 (T) solution, the membrane were further incubated for 1 h at room temperature with goat-rabbit IgG or goat-mouse IgG antibody coupled to horseradish peroxidase (GE Healthcare, Chicago, IL, USA) at 1:10,000 dilution in Can Get Signal Solution 2 (Toyobo, Osaka, Japan) and washed three times in TBS-T before detection. The expression of the proteins was detected by the ECL detection system (GE Healthcare, Chicago, IL, USA) using Chemi Doc Imaging Systems (Bio-Rad Laboratories, Hercules, CA, USA).

### 4.5. Chondrogenic Differentiation 

The chondrogenic differentiation test was conducted in accordance with previously published protocols [40]. ATDC5 cells were then incubated in the absence or the presence (12.5–50 μg/mL) of ECM components in DMEM/Ham′s F12 containing 5% FBS, penicillin/streptomycin, 10 µg/mL transferrin and 3 × 10^−8^ M sodium selenite at 37 °C in a humidified atmosphere of 5% CO_2_ in air for 21 days from post-confluence (7 days), without insulin, to evaluate chondrogenic differentiation. Insulin (10 μg/mL) was used as a positive control of chondrogenic differentiation. The medium containing ECM components or insulin was changed once every 2 or 3 days. The medium containing no ECM components or insulin as control was also changed once every 2 or 3 days.

### 4.6. Cartilage Calcification

The cartilage calcification test was conducted in accordance with previously published protocols [41]. ATDC5 cells were then incubated in the absence or the presence (10–250 μg/mL) of ECM components for 24 days as calcification-inducing conditions to evaluate cartilage calcification in αMEM medium containing 5% FBS, 10 μg/mL insulin, penicillin/streptomycin, 10 µg/mL transferrin and 3 × 10^−8^ M sodium selenite at 37 °C in a humidified atmosphere of 3% CO_2_ in air, after inducing chondrogenic differentiation in cells using insulin under the above conditions until day 28. The medium containing ECM components was changed once every 2 or 3 days. The medium containing no ECM components as a control was also changed once every 2 or 3 days.

### 4.7. RNA Isolation and Reverse Transcription (RT)-PCR 

Cultured ATDC5 cells were rinsed in PBS and total RNA was extracted using the RNeasy kit (QIAGEN, Hilden, Germany). The extracted RNA was quantified using a NanoDrop (Thermo Fisher Scientific, Waltham, MA, USA). cDNA was prepared from 50 ng of total RNA using the Prime Script RT reagent kit (Takara Bio, Kusatsu, Japan) for RT-PCR. The RT-PCR conditions were reverse transcription reaction at 37 °C for 15 min, and enzyme inactivation at 85 °C for 5 s.

### 4.8. Real-Time Quantitative PCR

Real-time quantitative PCR was performed using each specific primer and the SYBR Premix EX Taq II (Takara Bio, Kusatsu, Japan). Using the forward primer sequences 5′-3′, the reverse primer sequences 5′-3′ for the following targets: *Sox9* (using the forward primer sequences 5′-CATCACCCGCTCGCAATAC-3, the reverse primer sequences 5′-CCGGCTGCGTGACTGTAGTA-3), *Col2a1* (using the forward primer sequences 5′-CACACTGGTAAGTGGGGCAAGACCG-3′, the reverse primer sequences 5′-GGATTGTGTTGTTTCAGGGTTCGGG-3′), *aggrecan* (*Acan*) (using the forward primer sequences 5′-GAAATGACAACCCCAAGCAC-3′, the reverse primer sequences 5′-TCTCCGCTGATTTCAGTCCT-3′), *Igf-1* (using the forward primer sequences 5′-ACTGCTAAACACTTGGCAGGAG-3′, the reverse primer sequences 5′-TTGCAAGGTTTAAGGATACAGAGAC-3′), *Gdf5* (using the forward primer sequences 5′-GTAACAGCAGCGTGAAGTTGGAG-3′, the reverse primer sequences 5′-TTCCGTAAGATCCGCAGTTCAG-3′), *Bmp4* (using the forward primer sequences 5′-CTCTTCAACCTCAGCAGCATCC-3′, the reverse primer sequences 5′-TGGCAGTAGAAGGCCTGGTAGC-3′), *Mmp13* (using the forward primer sequences 5′-TGATGGACCTTCTGGTCTTCTGG-3′, the reverse primer sequences 5′-CATCCACATGGTTGGGAAGTTCT-3′), *Runx2* (using the forward primer sequences 5′-GCTTGATGACTCTAAACCTA-3′, the reverse primer sequences 5′-AAAAAGGGCCCAGTTCTGAA-3′), *Col10a1* (using the forward primer sequences 5′-ACTTCCTGTCAAGCTCATCC-3′, the reverse primer sequences 5′-TCCTGCATGTTTCCTAGATG-3′), *Mgp* (using the forward primer sequences 5′-CCTGTGCTACGAATCTCACGAA-3′, the reverse primer sequences 5′-TCGCAGGCCTCTCTGTTGAT-3′), *Ihh* (using the forward primer sequences 5′-CTCTCACAAGGCATGGGACAC-3′, the reverse primer sequences 5′-GGTCAGCCACAGCTGCAAAG-3′), *Pthrp* (using the forward primer sequences 5′-CAGTGGAGTGTCCTGGTATT-3′, the reverse primer sequences 5′-GATCTCCGCGATCAGATGGT-3′), *Pth1r* (using the forward primer sequences 5′-CTGTGGCAGATCCAGATGCACTA-3′, the reverse primer sequences 5′-GAAGTCCAATGCCAGTGTCCAG-3′) and *Rps18* (using the forward primer sequences 5′-TTCTGGCCAACGGTCTAGACAAC-3′, the reverse primer sequences 5′-CCAGTGGTCTTGGTGTGCTGA-3′), as the internal control, were purchased from Hokkaido System Science, Sapporo, Japan. Real-time fluorescence detection was performed using the Thermal Cycler Dice Real Time System Single (Takara Bio, Kusatsu, Japan). PCR cycling conditions were as follows, 94 °C for 15 min followed by 40 cycles at 94 °C for 30 s and 54–60 °C for 30 s.

### 4.9. Alcian Blue Staining

Cells were washed twice with PBS and fixed with 10% neutral buffered formalin for 30 min, after which, cells were stained with 1% Alcian blue 8GX (FUJIFILM Wako Pure Chemical Corporation, Osaka, Japan) in 0.1 M HCl overnight. After staining, cells were washed with 3% acetic acid three times and the images were acquired using phase-contrast microscopy (Olympus, Tokyo, Japan) and a digital camera. In the quantitative analysis, the ratio was calculated by selecting the stained area with Alcian blue by threshold processing from the whole image acquired with the same number of pixels by digital camera. The obtained whole images were quantified by WinROOF Ver.6.3.1 software (Mitani Co., Ltd., Fukui, Japan). Relative Alcian blue staining areas were normalized by control without ECM components.

### 4.10. Alizarin Red Staining

Cells were rinsed three times with PBS and fixed with methanol at –20 °C for 20 min. They were stained with 1% Alizarin red S (FUJIFILM Wako Pure Chemical Corporation, Osaka, Japan) at pH 6.4 for 5 min and washed with distilled water. The images were acquired using phase-contrast microscopy (Olympus, Tokyo, Japan) and a digital camera. In the quantitative analysis, the ratio was calculated by selecting the stained area with Alizarin red by threshold processing from the whole image acquired with the same number of pixels by digital camera. The obtained whole images were quantified by WinROOF Ver.6.3.1 software (Mitani Co., Ltd., Fukui, Japan). Relative Alizarin red staining areas were normalized by control without ECM components.

### 4.11. Histology of Chondrogenic Differentiation

ATDC5 cells for histology of chondrogenic differentiation were then incubated on cell culture insert in the absence or the presence (50 μg/mL) of sPG for 21 days from post-confluence without insulin. Insulin (10 μg/mL) was used as a positive control of chondrogenic differentiation. Samples were fixed in 4% paraformaldehyde, dehydrated, embedded in paraffin and sectioned. Then, 4–5 μm sections were stained with hematoxylin and eosin and examined under a light microscope.

### 4.12. Fluorescent Labeling of sPG

SPG was fluorescently labeled with ATTO 488 NHS ester (Sigma-Aldrich, St. Louis, MO, USA) for 2 h at room temperature. ATTO 488-labeled sPG was purified by Zeba desalting columns, 7K MWCO (Thermo Fisher Scientific, Waltham, MA, USA), with a 7 kDa molecular weight cut-off. 

### 4.13. Confocal Fluorescence Live Cell Imaging

ATDC5 or NHDF cells were incubated in the presence of ATTO 488-labeled sPG or ATTO 488 for 30 min. The cells were washed with PBS, then added with CellMask Deep Red (Thermo Fisher Scientific, Waltham, MA, USA) to recognize cell membrane and incubated for 30 min. The cells were also washed with PBS, followed by Live Cell Imaging with a LSM 710 CLSM (Carl Zeiss, Oberkochen, Germany) equipped with Temp Module and CO_2_ Module. The obtained optical sectioning (z-stack) was subjected to three-dimensional reconstruction using ZEN (Carl Zeiss, Oberkochen, Germany).

### 4.14. Statistical Analysis

The results were analyzed using JMP^TM^ 8 (SAS Institute Inc., Cary, NC, USA) software. The data were collected from at least three independent experiments and were expressed as the mean ± standard deviation (S.D.). For all results, assuming data in Gaussian distribution, data were analyzed by analysis of variance (ANOVA). The statistical comparisons between the different treatments were performed using Student′s t-test. To perform multiple comparisons, Dunnett′s or Tukey′s tests were used as post hoc tests after ANOVA. The statistical analysis of each result is described in the figure legends. In all analyses, a *p*-value < 0.05 was considered statistically significant. 

## 5. Conclusions

In this study, we showed that sPG confers a proliferative effect on chondroprogenitor cells, thereby both inducing chondrogenic differentiation, as well as inhibiting cartilage calcification. Importantly, these effects of sPG were more robust than those produced by HA, an established OA treatment. Our data suggest that application of sPG to cells induces a change in the cellular surface microenvironment, leading to the regulation of cell proliferation, differentiation and calcification, and it may confer a therapeutic benefit in treatment of OA.

## Supplementary Materials

Supplementary materials can be found at https://www.mdpi.com/1422-0067/21/20/7744/s1.

## Abbreviations

BMP	Bone morphogenetic protein
CLSM	Confocal laser scanning microscopy
CS	Chondroitin sulfate
ECM	Extracellular matrix
EGFR	Epidermal growth factor receptor
FGF	Fibroblast growth factor
GAG	Glycosaminoglycan
GDF5	Growth differentiation factor 5
GlcN	Glucosamine hydrochloride
HA	Hyaluronan
IGF-1	Insulin-like growth factor 1
IRS-1	Insulin receptor substrate 1
MGP	Matrix gla protein
OA	Osteoarthritis
PCM	Pericellular matrix
PG	Proteoglycan
PTHrP	Parathyroid hormone-related protein
Runx2	Runt-related transcription factor 2
SOX9	SRY-box transcription factor 9
TGF-β	Transforming growth factor beta
