# Supplementary material for: Exogenous Application of Proteoglycan to the Cell Surface Microenvironment Facilitates to Chondrogenic Differentiation and Maintenance
Source: Int J Mol Sci. 2020 Oct 19;21(20):7744. doi: 10.3390/ijms21207744 (PMC7589071; doi:10.3390/ijms21207744)
Supplement: Supplementary file 1 [file ijms-21-07744-s001.pdf]

Table S1: The disaccharide composition of commercial CS and purified GAG preparations mentioned in Results 2.1.

Sample	Proportion (%)				
	ΔDi-0S	ΔDi-6S	ΔDi-4S	ΔDi-diS$_D$	total
CS6M	2.3	57.7	24.5	15.5	100
CS4M	6.7	22.3	71.0	0.0	100
Purified GAG	12.2	60.6	26.6	0.6	100
